# Clonal Populations of Amniotic Cells by Dilution and Direct Plating: Evidence for Hidden Diversity

**DOI:** 10.1155/2012/485950

**Published:** 2012-09-11

**Authors:** Patricia G. Wilson, Lorna Devkota, Tiffany Payne, Laddie Crisp, Allison Winter, Zhan Wang

**Affiliations:** ^1^Wake Forest School of Medicine, Institute for Regenerative Medicine, Medical Center Blvd., Winston-Salem, NC 27157, USA; ^2^Wake Forest University, Medical Center Blvd., Winston-Salem, NC 27157, USA; ^3^Wake Forest School of Medicine, Medical Center Blvd., Winston-Salem, NC 27157, USA

## Abstract

Fetal cells are widely considered a superior cell source for regenerative medicine; fetal cells show higher proliferative capacity and have undergone fewer replicative cycles that could generate spontaneous mutations. Fetal cells in amniotic fluid were among the first normal primary cells to be cultured ex vivo, but the undefined composition of amniotic fluid has hindered advance for regenerative applications. We first developed a highly efficient method to generate clonal populations by dilution of amniocentesis samples in media and direct plating without intervening refrigeration, centrifugation, or exposure of cells to the paracrine effects in mixed cell cultures. More than 40 clonal populations were recovered from 4 amniocentesis samples and representative clones were characterized by flow cytometry, conventional assays for differentiation potential, immunofluorescence imaging, and transcript analysis. The results revealed previously unreported diversity among stromal and epithelial cell types and identified unique cell types that could be lost or undetected in mixed cell populations. The differentiation potential of amniotic cells proved to be uncoupled from expression of definitive cell surface or cytoplasmic markers for stromal and epithelial cells. Evidence for diversity among stromal and epithelial cells in amniotic fluid bears on interpretations applied to molecular and functional tests of amniotic cell populations.

## 1. Introduction

The mission of regenerative medicine is to repair or replace tissues and organs that have been damaged by trauma, disease, or aging with living bioengineered tissue that restores function [1, 2]. Among cell sources for regenerative applications, stromal cells have gained increasing interest. Stromal cells, also known as multipotent stromal cells or MSCs, have been isolated from virtually all adult and postnatal tissues and organs [3]. Recent studies focus on use of stromal cells as a cell-based delivery system for trophic factors to repair damage and immunomodulatory activities to suppress damaging effects of inflammation, autoimmunity, and graft-versus-host disease (GVHD) that can cause rejection of transplanted organs and tissues [4–7]. Fetal MSCs may be superior to other sources of MSCs with respect to proliferation capacity [8, 9]. Fetal cells can be obtained with minimally invasive methods during routine amniocentesis [10] and easily transitioned to ex vivo culture [11]. However, amniocentesis samples are complex mixtures of cells that are sloughed from fetal and placental surfaces that are exposed to amniotic fluid [12, 13]. Standards for cell-based therapies require cell populations that satisfy criteria for safety and efficacy [14–16]. Incorporation of amniotic cells into regenerative applications would be advanced by a better understanding of the complexity within amniotic cell populations and variation among amniocentesis samples from different donors.

Similarities and differences among amniotic cells have been classified to a large extent on cell shape. Clones of amniotic cells were first isolated almost 4 decades ago with cloning rings and classified on the basis of colony morphology, see Hoehn and Salk (1982) for a contemporaneous review [11]. F-type colonies consisted of “spindle-” shaped fibroblast-like cells forming dense, multilayered colonies that are reminiscent of confluent stromal cell cultures. E-type colonies were formed by “epithelioid” cells with smooth margins and juxtaposed cell boundaries. AF-type colonies were the most common colony type from amniotic fluid, representing ~70% of colonies in one study, and considered to be specific to amniotic fluid. AF colonies consisted of fibroblast-like cells in a radial arrangement surrounding a dense amorphic cell aggregate that was resistant to enzymatic methods to generate single cells. While it is unclear whether AF-colonies reflect a cell type unique to amniotic fluid or the cell culture methods used, these pioneering studies set the stage for ex vivo culture of cells from amniotic fluid and provided the first widely used source of normal, rather than transformed, cells for biomedical research.

Current criteria for evaluating stromal cell identity and function have been based on bone-marrow-derived MSCs. These BMMSCs are the best studied stromal cells and are currently in clinical trials for treatment of several pathologies (http://www.clinicaltrials.gov/). BMMSCs are derived from bone marrow aspirates and adhere to plastic culture wares, in contrast to hematopoietic derivatives of bone marrow that proliferate in suspension [17]. The International Society for Cellular Therapy has established criteria for assigning BMMSC identity [18, 19], including adherence to plastic, differentiation into mesenchymal lineages of fat, bone, and cartilage, and expression of the cell surface markers endoglin or CD105, ecto 5′ nucleotidase or CD73, and Thy-1 or CD90. More than 95% of cells must express these markers, although the acceptable levels of absolute or relative expression have not been established. The relationship between these criteria for stromal cell identity and the potential therapeutic properties of stromal cells is not clear, in part because expression is correlative rather than causative and because the definitive set of cell surface antigens is not unique to stromal cells. Outstanding questions include whether stromal cell populations vary in expression of BMMSC-definitive markers and whether expression profiles are predictive of differentiation potential.

The diversity of cells in amniotic fluid bears on interpretation of molecular analyses and functional tests since the outcomes could reflect additive effects of one or more cell types. The presence of both epithelial and stromal cells in amniotic cell cultures has been reported in most [20–22] but not all studies [11, 23, 24]. Epithelial cells have been noted to quickly disappear during propagation of mixed cell cultures [25]. Amniotic cell cultures acquire a uniform stromal cell appearance [26] that could reflect replicative senescence of epithelial cells [11]. Loss of epithelial cells in culture could also reflect epithelial to mesenchymal transition (EMT), a molecular pathway in which epithelial cells become stromal cells with the immunoreactive profile and differentiation potential that is expected of MSCs [27, 28]. The complexity of amniotic cell populations could be addressed through analysis of clonal populations. Clonal populations of amniotic cells have been established with cloning rings [20], immunoisolation of cells expressing the receptor for Steele Factor or CD117 [29], enzymatic treatment of preestablished cultures to generate single cells followed by limiting dilution [24, 29], and variations on these methods [30]. In each case significant effort is required to generate clones and amniotic cells are exposed to paracrine signaling in mixed cell populations during isolation.

The initial goal of this work was to develop an efficient method to establish independent clones from uncultured amniocentesis samples with minimal manipulation and without ex vivo expansion in mixed cell populations. We further asked whether clonal populations of stromal and epithelial cells differed from each other and from BMMSCs. Clones were characterized by phase microscopy, flow cytometry, in vitro differentiation, and high-resolution immunofluorescence imaging. The results revealed phenotypically and functionally distinct stromal cell clones and, for the first time, clonal populations of long-lived epithelial cells. Our findings show that the differentiation potential of amniotic cells need not mirror expression of cell surface markers of other amniotic cell clones or expression profiles of BMMSCs. We show that clones of amniotic stromal cells and epithelial cells can share nearly indistinguishable profiles of cell surface markers and coexpress cytoplasmic markers for epithelial and stromal cells, but differ in adipogenic and osteogenic potential. Analysis of multiple nonclonal mixed cell populations from different donors by high-resolution imaging identified most, but not all, of the same cell types in clonal populations and revealed clear differences between mixed cell populations. Taken together, our results shed new light on the differences among amniotic cells and raise questions of their source.

## 2. Materials and Methods

### 2.1. Cell Culture

Amniocentesis samples were donated with informed written consent, approved by the Institutional Review Board of Wake Forest University Health Sciences (2008) and deidentified for research. The age of the mother, period of gestation at which amniocentesis was performed, or the results of genetic testing were not approved for disclosure.

All amniocentesis samples were maintained at room temperature prior to cell culture. Samples used to derive mixed cell populations were diluted 1 : 1 to 1 : 2 in serum-containing media and plated in 1 or 2 wells of a 6-well plate. Samples used to derive clonal populations were diluted as described in Table 1 and distributed among one or more 24-well plates. Media and any nonadherent cells were routinely combined with approximately one-half volume of fresh media, transferred into new plates after 48–72 hrs, and discarded after another 72 to 96 hrs, allowing cells a total of 5 days to 7 days to adhere to culture wares. Cells were routinely maintained in Chang's Media, which contained *α*-MEM supplemented with 15% FBS, 1% glutamine and 1% penicillin/streptomycin, 18% Chang B, and 2% Chang C (Irvine Scientific). All cell cultures were maintained at 37°C with 5% CO_2_ in humidified incubators. Media components were obtained from Gibco/Invitrogen unless stated otherwise. Tissue culture wares (BD Falcon) were not pretreated with extracellular matrix proteins except for plates used in assays for differentiation potential as detailed below. 

Primary subconfluent cultures were passaged as needed to maintain healthy populations. Cells were passaged with Accutase diluted 1 : 4 in calcium and magnesium-free Dulbecco's phosphate buffered saline (DPBS) with standard methods. Clonal populations in a single well of a 24-well plate were maintained as independent lines. Populations were passaged for expansion into the next largest culture volume: from 24 wells to 35 mm wells or to 60 mm plates before expansion into 100 mm plates and/or cryopreservation. Mixed cell populations that were derived from a single sample were harvested with enzymatic treatments, pooled, and cryopreserved in multiple aliquots with standard methods for long-term storage in liquid nitrogen.

BMMSCs were expanded from cryofrozen mononuclear cells that were derived from human bone marrow (Lonza, 2 M-125 C). Cryovials were thawed in *α*-MEM media supplemented with 10% heat-inactivated FBS, 1% glutamine, and 1% penicillin/streptomycin in 15 cm treated culture dishes. Some cells attached after several days and unattached cells were discarded in the first media change. The attached cells were then expanded in Chang's media which better supported continued growth and simplified media preparation. BMMSCs at an early passage were frozen in small aliquots and thawed as needed. 

### 2.2. Imaging and Immunocytochemistry

Cells were propagated in multiwell tissue culture plates on cover glass or in multiwell Permanox chamber slides (Nunc) for immunostaining. Samples were washed with Dulbecco's phosphate buffered saline (DPBS) and fixed for ~20 min with 2% paraformaldehyde (EM Sciences) in DPBS. Fixative was made fresh by dilution of 16% paraformaldehyde and frozen in aliquots at –80°C. Aliquots of the fixative were thawed when needed, used once, and then discarded. Following fixation, cells were briefly washed twice in DPBS with or without ~ 0.1% (v/v) Triton-X and 0.1% (v/v) Tween-20 (Sigma) for permeabilization as needed. Cells were routinely blocked for ~30 min in blocking buffer containing 3% bovine serum albumin (BSA fraction IV, Jackson Immunolabs) in DPBS with or without detergents as appropriate. Cells were incubated with primary antibodies in blocking buffer at room temperature (RT) for ~1 hr and then washed for a total 30 min. Secondary antibodies in blocking buffer were applied for at least one hr at RT or overnight at 4°C. Primary antibodies for immunofluorescence were used at the following dilutions: AE1/AE3 (1 : 100) DAKO; vimentin (1 : 100) and fibronectin (1 : 100) Santa Cruz, N-cadherin (1 : 100) Pharmingen. Alexa Fluor conjugated secondary antibodies (1 : 1,000) were obtained from Invitrogen/Molecular Probes. 

Stained samples were washed as before and mounted in Prolong Gold Plus (Molecular Probes) mounting media containing 4′,6-diamidino-2-phenylindole (DAPI) for viewing. Unless noted otherwise, wide-field images were captured with Image-Pro software using a QImaging CCD camera mounted on a Leica upright microscope using a 20X dry objective (NA 0.40) and imported into Photoshop for presentation. Immunostaining was repeated in at least 2 technical replicates and in more than 3 independent trials for each marker/combination tested. The images shown throughout this paper are representative; conclusions were based on at least 3 fields of view for each replicate and inspection of more than 100 cells.

### 2.3. Flow Cytometry

Cells for flow cytometry were enzymatically treated with Accutase to generate single-cell suspensions. Cells were collected by centrifugation at 300 × g for 5 min, washed once in DPBS, and collected again by centrifugation. Cell pellets were resuspended in approximately 200 *μ*l of DBPS before immediate dilution into 20 ml of 2% paraformaldehyde in DPBS. Cells were fixed for 20 min with gentle rocking, collected by centrifugation as described above, washed once in DPBS, and either used immediately or stored at 4°C. Prior to staining for flow cytometry, cells were collected by centrifugation and then resuspended in blocking buffer for 30 min. Cell suspensions in blocking buffer were filtered with 40 *μ*m mesh baskets to eliminate cell clumps and then dispensed into individual 15 ml conical tubes for immunostaining. Cells were stained with fluorochrome-conjugated mouse monoclonal antibodies or with matched isotype control antibodies in blocking buffer for at least 1 hr at room temp. Cells were then washed twice with DPBS and resuspended in ~300 *μ*l blocking buffer for analysis by flow cytometry.

Fluorochrome-conjugated antibodies were diluted in blocking buffer as recommended by the vendor: CD90 Fluorescein isothiocyanate or FITC (Millipore), CD90 Allophycocyanin or APC (BD Pharmingen), CD105 Alexa Fluor 647 (BD Pharmingen), CD105 FITC, (BD Pharmingen), CD73 APC (BD Pharmingen), SSEA4 Alexa Fluor 647 (BD Pharmingen), CD44 FITC (BD Pharmingen), and CD29 Alexa Fluor 488 (Molecular Probes/Invitrogen). Isotype controls were obtained from BD Pharmingen. Populations were gated to include only live cells and to exclude background staining that could be attributed to nonspecific staining by isotype antibody controls. Data was based on detection of 10,000 events for each marker. Data collection was performed with a FACSCalibur (BD Biosciences) flow cytometer and the results were imported into FlowJo 7.6.4 for analysis and presentation.

### 2.4. Adipogenic and Osteogenic Differentiation

Healthy cells were cultured to ~90% confluence in Chang's media on cultureware pretreated with a 1 : 300 dilution of growth-factor-reduced Matrigel. Media were then switched to *α*-MEM supplemented with 10% FBS and agents for osteogenic [0.1 *μ*M dexamethasone, 10 mM *β*-glycerol phosphate, 50 *μ*M ascorbic acid 2-phosphate] or adipogenic [1 *μ*M dexamethasone, 5 *μ*g/ml insulin, 0.5 mM isobutylmethylxanthine (IBMX), 60 *μ*M indomethacin] differentiation [31–33]. Media without differentiation supplements were used as a negative control and all media were exchanged every 3–5 days. Osteogenesis and adipogenesis were assessed with alizarin red and oil red-O staining, respectively, using standard histochemical methods. Each cell population was differentiated in at least 2 trials with more than 3 technical replicates, using undifferentiated cells in the same culture plate for controls and BMMSCs for negative and positive controls, respectively.

### 2.5. Transcript Analysis

Total RNA was extracted with RNAeasy kits (Qiagen) with DNAse treatment to eliminate genomic DNA according to manufacture directions. RNA was converted to cDNA with Superscript III First Strand Synthesis kits (Invitrogen). TaqMan gene expression assays were used to detect transcripts of glucuronidase-*β* (GUSB), Hs00939627, E-Cadherin (CDH1) Hs01023894, and N-cadherin (CDH2), Hs00983056. Ct values of replicate assays were averaged and averages were normalized to expression of GUSB, an internal control that was included in all experiments.

## 3. Results

### 3.1. Dilution and Direct Plating of Uncultured Amniocentesis Samples

Centrifugation is commonly used to concentrate cells in amniotic fluid and minimize dilution of media, but it was unclear whether this manipulation is necessary to recover viable amniotic cell populations. Newly isolated amniotic fluid samples of less than 2.0 ml were diluted 1 : 1 or 1 : 2 in serum-containing media and directly plated in individual wells of a 6-well tissue-culture-treated plate. As early as the first 48 to 72 hrs of plating, a few adherent cells were detected in each sample. These populations, designated as ChM mixed cell populations, were expanded into larger culture wares within a few days. Virtually all primary cultures expanded as monolayers. Subsequent passaging with trypsin generated colonies with centrally located aggregates, reminiscent of descriptions of AF-type colonies in seminal works [11]. With rare exceptions, cultures passaged with Accutase expanded as monolayers without aggregate formation, suggesting that aggregate formation may reflect cell culture methods rather than a specific feature of a unique class of cells in amniotic fluid [11]. These findings together indicated that cell concentration is unnecessary for recovery of proliferating cells and that reducing the concentration of serum by ~1 : 1-2 does not preclude cell attachment and proliferation.

We next tested whether multiple independent populations could be isolated simply by diluting amniocentesis samples into larger volumes of media. The four subsequent samples that we received were diluted in 25 to 125 mL of serum-containing media and plated into one or more 24-well plates treated for tissue culture. Media and nonadherent cells were transferred as replicates to new plates after 48 hr to 72 hr. The 4 amniocentesis samples RC, LB, GW, and PB generated 12, 9, 14, and 9 independent viable cell populations, respectively (Table 1). Each population was assigned a name reflecting its source and its isolation address: the sample (RC, LB, GW, PB), primary or secondary (2) transfer, plate number (1–5), row (A–D), and column (1–6). Similar numbers of cell populations were derived from the 4 samples that we tested, but the sample size (*n* = 4) precludes meaningful tests of statistical significance. Nonetheless, 44 independent viable cell lines were generated within 2 to 4 days of plating less than 8 mls of uncultured amniocentesis sample using this simple and highly efficient method.

### 3.2. Clonal Populations Expand from a Discrete Point Source

**Figure 1:**
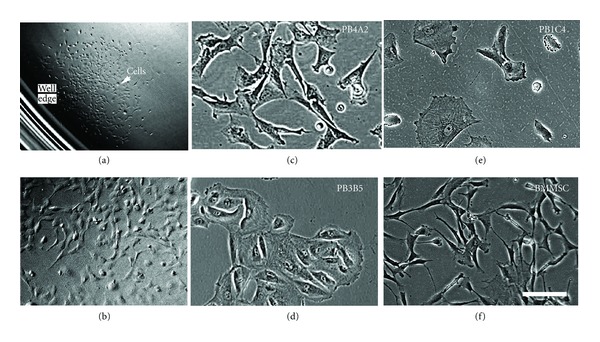
Clonal population of cells from amniocentesis samples. Uncultured amniocentesis samples were diluted into growth media and plated into 24-well tissue-culture-treated plates. (a) Low-magnification image shows an expanding clonal population in one well of 24-well plate as a spherical cluster that is located near the well edge. Arrow indicates cells. (b) Higher magnification (5x) of the same population shows apparently well isolated cells. Representative clonal populations are shown here with passage numbers (p) indicated: (c) PB4A2p4, (d) PB3A5p3, (e) PB1C4p11, and (f) BMMSCp5 control cells. Stromal (c) PB4A2p4 and (e) PB1C4p11 cell populations resemble (f) BMMSCp4 cells, showing irregular cell boundaries while (d) PB3B5 epithelial cells were typically spheroid and often found in clusters with closely apposed boundaries. Scale bar, 100 microns. Magnification is identical in (c), (d), (e), and (f).

Cell populations developed as expected of single-cell clones; daily monitoring by phase microscopy showed continual expansion of a small spherical cluster of cells, most often at the edge of the well (Figures 1(a), and 1(b)), but occasionally near the well center. We did not detect dispersion of cells throughout wells during population expansion, suggesting that any cell migration was limited to short distances. The clonal character of cell populations was consistent with detection of 2-well-separated expanding spheres in single wells, as if each cluster was initiated from 2 different founder cells. On the basis of these observations, we designate individual populations arising from a single spherical cluster as clonal populations and only such populations were studied further.

Expanded clonal populations could be classified by phase microscopy on the basis of cell morphology, designated here as stromal or epithelial to use terminology that is widely applied in cell biology and consistent with descriptions of cells in mixed cell populations that were established by others [11, 34, 35]. Stromal cell populations were dominated by large, well-separated flat cells with multipolar morphology and irregular cytoplasmic extensions (Figures 1(c), and 1(e)) that resembled those of BMMSCs (Figure 1(f)). Stromal cell populations typically contained very small cells with very little cytoplasm and small masses of cytoplasm without detectable nuclei. Epithelial populations consisted of spherical cells with a centrally located nucleus (Figure 1(d)), appearing as a phase refractive dome surrounded by flattened phase transparent cytoplasm. Primary populations of epithelial cells tended to be juxtaposed to each other in islands of multiple cells. All of the amniocentesis samples produced at least one apparently senescent population that did not expand enough to passage or failed to proliferate after passaging. While we did not attempt to expand and characterize all populations, simple inspection during colony expansion indicated that approximately half of the clones classified (*n* = 22) were stromal and half were epithelial. Although AF-type clones fitting the description of Hoehn and Salk [11] were not typical, one GW clone consistently developed aggregates during repeated passages for reasons that are not yet clear. These findings show that morphologically distinct clonal populations can be isolated from uncultured amniocentesis samples simply by dilution and direct plating of fresh uncultured samples.

### 3.3. Analysis of Cell Surface Marker Expression by Flow Cytometry

**Figure 2:**
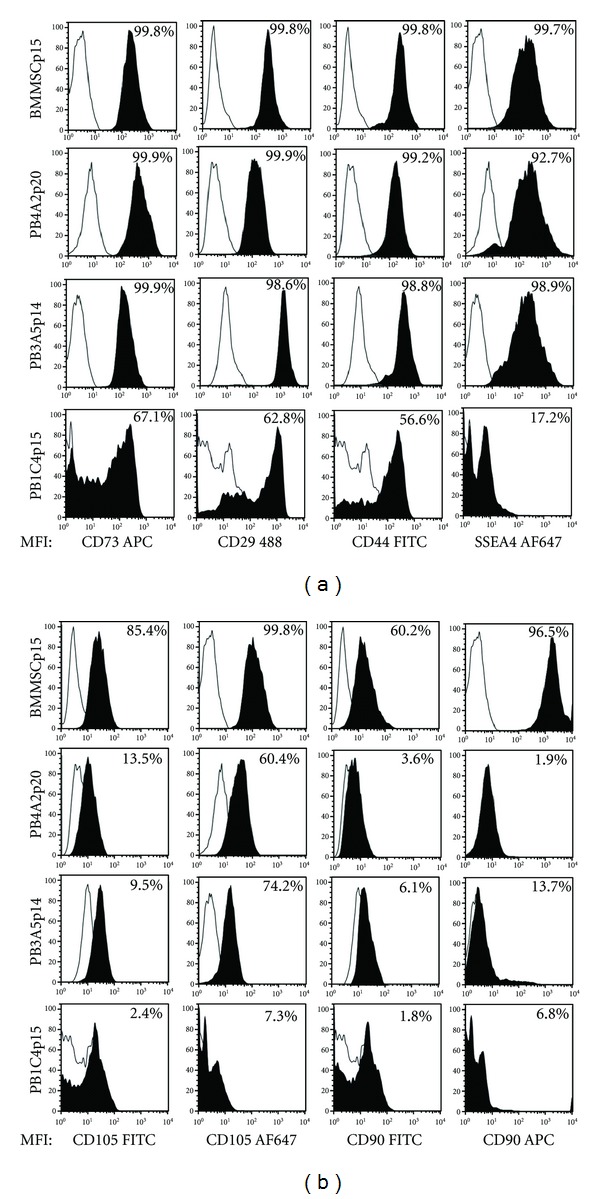
Flow cytometry of cell surface markers. Cell populations BMMSCp5, PB4A2p20, PB3B5p14, and PB1C4p15 were stained with antibodies against (a) CD73, CD29, CD44, SSEA4 and (b) against CD90 and CD105. Cell populations are indicated in vertical text on the left of the corresponding row of histograms for each marker. The markers and conjugated fluarochromes are indicated at the bottom of the corresponding column of each histogram. The *x*-axis of all histograms corresponds to the mean florescence intensity (MFI) in log scale. The *y*-axis is the percentage (%) of events in linear scale that were detected at each position of MFI along the *x*-axis. Cell populations were gated to exclude presumptive dead cells and debris. The histogram of isotype controls is depicted by black line and the marker that is assayed is depicted in the filled histogram. 10,000 events were scored for all populations. The percentage of immunostained cells, excluding those stained by isotype controls, is indicated within each histogram.

Expression of cell surface markers was tested by flow cytometry to determine whether the profiles of amniotic stromal cell clones were comparable to those of BMMSCs and to determine whether flow cytometry could be distinguished between clonal populations of stromal and epithelial cells. Cells were immunostained with antibodies against CD73, CD90, and CD105, the minimal set of cell surface markers that are required for assignment of BMMSC identity [18] as well as additional markers that are widely used to characterize MSCs [29], including integrin*β*1 that is detected with CD29 antibodies, the CD44 glycoprotein receptor for hyaluronic acid, and the stage-specific embryonic antigen 4 (SSEA4). Profiles were first established for BMMSCs as a positive control for immunodetection procedures. The results showed that nearly all BMMSCs expressed CD73 as well as CD29, CD44, and SSEA4 (Figure 2(a)). Large subsets of BMMSCs expressed CD105 and CD90, ~86% and 60% respectively, using FITC-conjugated antibodies (Figure 2(b)), but the proportion was less than the 95% frequency expected [36]. The proportion of CD105 and CD90 immunopositive cells increased to 100% and 97%, respectively, with antibodies conjugated to the long wavelength flours Alexa Fluor 647 (AF647) and allophycocyanin (APC) that increase the sensitivity of detection (Figure 2(b)). Both sets of FITC- and APC/AF647-conjugated antibodies were used in the remaining experiments as an added measure of confidence for expression of CD90 and CD105 in amniotic cell populations.

Clones from the PB amniocentesis sample were analyzed: two stromal cell clones, PB4A2 and PB1C4, and an epithelial cell clone PB3B5. Like BMMSCs, nearly all PB4A2 and PB3B5 cells expressed CD73 as well as CD29, CD44, and SSEA4 (Figure 2(a)). PB4A2 stromal cells and PB3B5 epithelial cells showed very similar profiles of CD105 and CD90 expression; less than ~14% and ~7% of these cell populations expressed CD105 and CD90, respectively, as assayed with FITC-conjugated antibodies. The proportion of CD105 immunopositive PB4A2 and PB3B5 cells rose to 60% and 75%, respectively, with AF647-conjugated antibodies, but CD90 detection showed little change. In comparison with BMMSCs that were run in parallel, the mean fluorescence intensity (MFI) associated with immunostaining of CD105 and CD90 on PB4A2 stromal cells and PB3B5 epithelial cells was only modest or extremely low, indicating that CD105 and CD90 are not highly expressed. Two conclusions can be drawn from these findings. First, expression of CD29, CD44, CD73, and SSEA4 did not distinguish between BMMSCs and clones of PB4A2 stromal cells and PB3B5 epithelial cells. Second, flow cytometry did not distinguish between populations of PB4A2 stromal cells and PB3B5 epithelial cells. Finally, CD105 and CD90 expression in both PB4A2 stromal cells and PB3B5 epithelial cells was notably lower than in BMMSCs.

PB1C4 stromal cells differed from other cell populations; 67%, 63%, and 57% of cells expressed CD73, CD29, and CD44, respectively, and only ~17% of cells expressed SSEA4 (Figure 2(a)). Fewer than 8% of PB1C4 stromal cells were immunopositive for either CD105 or CD90, even with AF647/APC-conjugated antibodies. Finally, the antibodies tested generated a single major peak in signal intensity in all other populations, but profiles of both control and test populations of PB1C4 cells showed multiple major peaks and a broad range of signal intensities. Although the molecular basis for the differences between PB1C4 and other cell populations is not clear, these data together with the data above show that dilution and direct plating can generate distinct clonal populations of stromal cells.

### 3.4. Osteogenic Differentiation

**Figure 3:**
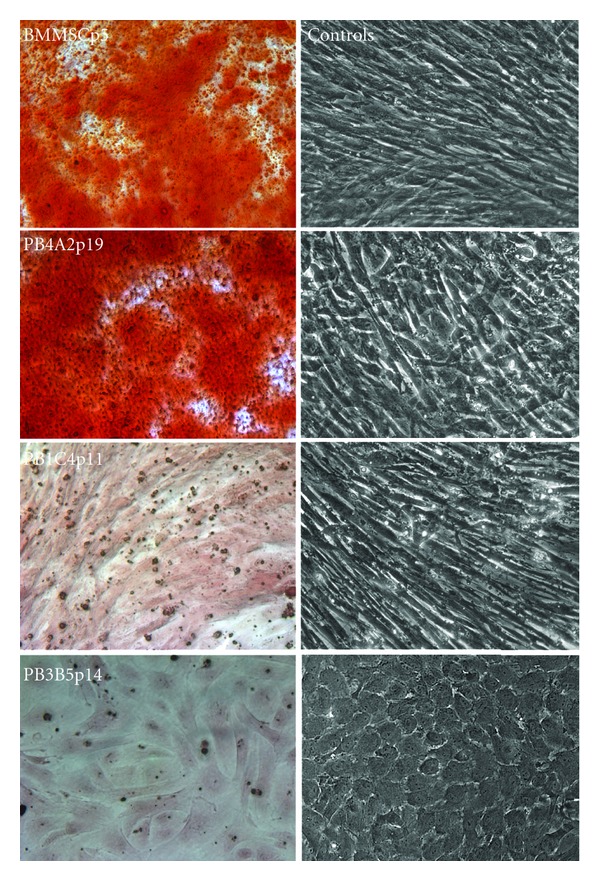
Osteogenic differentiation with companion control populations. Populations of BMMSCp5, PB4A2p19, PB1C4p11, and PB3B5p14 cells were expanded in growth media and then maintained in osteogenic media for 3 to 4 weeks. Robust deposition of calcium, which stains with alizarin red, was generated in BMMSCs and PB4A2 cell populations, but not in PB1C4 or PB3B5 cell populations. The corresponding populations in control media without differentiation supplements are shown in the column on the right. Note the cross-hatched appearance of overly confluent populations of BMMSCp5, PB4A2p19, and PB1C4p11 cells while PB3B5p14 cell populations that apparently ceased proliferation near confluence. Magnification is identical in all panels.

Results from flow cytometry suggested that amniotic cells would not show differentiation given that cell surface marker expression differed significantly from BMMSCs. Clonal cell populations were tested for osteogenic differentiation using standard methods. Subconfluent cultures of PB4A2p19, PB3B5p14, PB1C4p11, and BBMSCp5 cells were seeded in multiwell plates at near-confluent densities. Cells were subsequently maintained in differentiation media for 3 to 4 weeks, exchanging media every 3 to 5 days. Cells in media without differentiation supplements were used as negative controls. Following fixation and staining with alizarin red, robust deposition of calcium was detected in both BMMSCs and PB4A2 populations, but neither PB1C4 cells nor PB3B5 cells showed calcium deposition (Figure 3). Together with the results of flow cytometry, differential osteogenic potential of PB4A2 and PB1C4 populations supports recovery of distinct clones by dilution and direct plating. Further, the absence of robust expression of CD90 and CD105 suggests that expression of these markers is not predictive of osteogenic potential.

### 3.5. Adipogenic Differentiation

**Figure 4:**
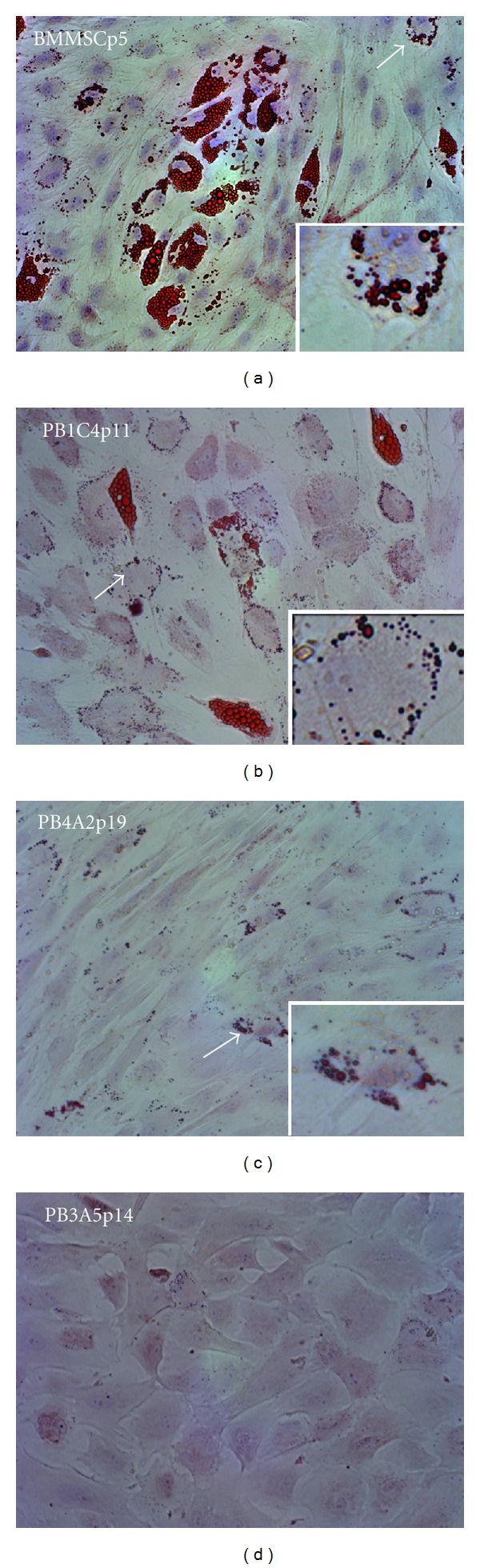
Adipogenic differentiation. Populations of BMMSCp5, PB4A2p19, PB1C4p11, and PB3B5p14 cells were maintained in adipogenic media for 3 to 4 weeks. Representative bright field images show robust adipogenic differentiation in BMMSCs and PB1C4 cell populations as indicated by oil red-O stained spheres. Arrows and inserts indicate very small oil red-O positive spheres in BMMSC, PB4A2, and PB1C4 populations that were not detected in PB3B5 populations. Magnification is identical in all panels.

Parallel experiments tested for adipogenic differentiation using standard methods of differentiation followed by staining with oil red-O to detect fat droplets (Figure 4). Phase microscopy showed the appearance of fat droplets accumulating in BMMSCs and PB1C4 cells within 2 weeks after induction (data not shown). Staining with oil red-O showed large bright red droplets in approximately 30% of BMMSCs and approximately 10% of PB1C4 cells. Although PB4A2 populations showed only occasional cells (<1%) with similar clusters of large droplets, approximately 10% to 30% of PB4A2 cells had clusters of small bright oil red reactive droplets. Similar clusters of small oil red-O reactive droplets were also present in BMMSCs and PB1C4 cells. PB3B5 cells did not show oil red-O reactive droplets, indicating that PB3B5 cells do not have adipogenic potential and that small oil red-O reactive droplets are not artifacts of staining. Taken together with the evidence for osteogenic potential, these findings indicate that clonal populations of PB4A2 and PB1C4 cells have distinct differentiation potentials. 

### 3.6. Immunostaining for Intermediate Filaments Typical of Epithelial and Stromal Cells

The results thus far showed differentiation of stromal, but not epithelial, cell populations. We next asked whether differentiation potential could be correlated with expression of stromal rather than epithelial cell markers. Cell populations were immunostained with panantibodies against keratins, a superfamily of intermediate filament family proteins that is expressed in epithelia [37], and vimentin, another member of intermediate filament superfamily that is widely used as a marker for stromal cells. BMMSCs cells showed well-organized immunopositive vimentin filaments as would be expected for stromal cells, but only a low level of diffuse keratin staining was detected in BMMSCs that was attributed to background staining. PB4A2 and PB3B5 cell populations showed bright immunopositive networks of both vimentin and keratin although the intensity of keratin immunostaining showed more variation in comparison to that of vimentin. In contrast to the other populations tested, the clonal population of PB1C4 cells did not show immunostaining of either vimentin or keratin networks. These findings show that expression of keratins or vimentin did not correlate with osteogenic and adipogenic differentiation potential. Further, amniotic stromal cells can be discriminated from one another on the basis of keratin and vimentin networks.

### 3.7. Immunodetection of Fibronectin and N-Cadherin

The absence of vimentin networks raised the question of whether PB1C4 stromal progenitors expressed other stromal cell markers. Fibronectin is an extracellular matrix (ECM) protein that is highly expressed in stromal cells, although it is not exclusive to these cells [38]. N-cadherin is a cell adhesion molecule that is expressed in mesenchymal cells [39], in contrast to E-cadherin which is highly expressed in epithelial cells [28]. Double labeling experiments showed coexpression of fibronectin and N-Cadherin in all populations tested (Figure 6). However, differences were detected; BMMSCs showed elaborate networks of fibronectin while PB4A2 and PB3B5 cells showed sparse fibronectin filaments except localized areas of high cell density. PB1C4 cells showed dramatic immunostaining of fibronectin filaments, even in low-density cell cultures. Fibronectin filaments in PB1C4 cells appeared as almost parallel arrangements of short filamentous structures that were reminiscent of porcupine quills in contrast to the cross-hatched networks of long filaments in BMMSCs. These results together with the results of flow cytometry and differentiation assays indicate that PB1C4 cells represent a unique clonal population of stromal cells that was isolated by dilution and direct plating.

### 3.8. Differential Expression of Vimentin and Keratins in Mixed Cell Populations

Variation among amniotic cell clones predicted that ChM mixed cell populations would contain that same mixture of epithelial and stromal cell types. We tested this prediction using several of the ChM mixed cell populations that we isolated from different donors at the onset of this study. Preliminary inspection by phase microscopy indicated that ChM populations varied in the apparent proportion of epithelial and stromal cells; epithelial cells were highly enriched in the ChM1 population, but few were detected in other ChM populations (data not shown). Immunostaining with antibodies against keratin and vimentin or keratin and fibronectin revealed diversity in the size and shape of cells within and between ChM populations. The ChM1 population contained many large spherical cells with prominent networks of keratin and vimentin, representing 85% of cells (*n* > 200) as well as smaller cells with more irregular cell shapes (Figure 7(a)–7(d)). ChM populations also contained cells that were immunopositive for vimentin filaments, but immunonegative for keratin, showing only diffuse background staining. These observations showed that amniocentesis samples contain a mixture of stromal and epithelial cells, including some of the same cell types isolated in clonal populations by dilution and direct plating.

Immunostaining for fibronectin and keratin revealed essentially ubiquitous fibronectin staining (Figures 7(e) and 7(f)), although dense cultures showed areas of elaborate networks that resembled fibronectin networks in BMMSCs (data not shown). ChM1 populations contained large spherical cells with striking umbrella-like arrangements fibronectin quills and prominent networks of keratin (Figure 7(e)). Although the short quills of fibronectin in epithelial cells in mixed cell populations were similar in appearance, we did not detect cells that were similar to PB1C4 cells in any of the populations tested. These findings suggest that PB1C4 cell types may be rare or difficult to detect in mixed cell populations.

### 3.9. Epithelial Cell Populations Vary in Expression of E-Cadherin and N-Cadherin Transcripts

Coexpression of stromal and epithelial cell markers by immunofluorescence (Figure 6) raised the question of whether PB3B5 epithelial cells expressed transcripts of E-cadherin. Although all of the tested clones were immunopositive for N-cadherin, PB3B5 cells were immunonegative for E-cadherin (data not shown) as expected of epithelial cells. Sensitive gene expression assays were used to test for E-cadherin and N-cadherin transcripts in clonal populations as well as in the ChM1 mixed cell population. Primary cultures of BMMSCs and epithelial cells derived from human urothelium were used as controls for stromal and epithelial cells, respectively. N-Cadherin transcripts were detected in all control and amniotic cell populations; however E-cadherin transcripts were only detected in ChM1 cells and in control uroepithelial cells (Table 2). E-Cadherin expression in both control and ChM1 cell populations was confirmed by immunofluorescence analysis (data not shown). These findings show diversity among amniotic epithelial cells that can be defined by the presence or apparent absence of E-cadherin expression.

## 4. Discussion

### 4.1. Isolation of Phenotypically Distinct Clonal Populations by Dilution and Direct Plating

This work provides proof of concept that dilution of amniocentesis samples and direct plating, without refrigeration or centrifugation, is a highly efficient method to generate unique clonal populations. Clonal identity of PB4A2, PB3B5, and PB1C4 populations is reflected in the phenotypic differences among these clonal lines (Table 3). One inference of our findings is that cell populations in amniotic fluid have greater diversity than can be appreciated by assignment of amniotic cells as epithelial or stromal cell types on the basis of morphology by phase microscopy. 

Dilution and direct plating allow efficient recovery of cell types that might otherwise be lost or undetected in mixed cell populations. This view is supported by 2 observations. First, we isolated clones of long-lived epithelial cells; PB3B5 cells have been in culture for more than 25 passages. This is significant because others have noted that clonal populations of epithelial cells are difficult to maintain beyond 5 or 6 passages [35, 40, 41] and that amniotic cell cultures either show or acquire a uniform stromal or fibroblast-like morphology during culture [26, 42–44]. Long-lived clonal populations like PB3B5 could reflect unique epithelial cell types and/or propagation of epithelial cell clones without the paracrine effects that may be present in mixed cell populations. A second observation supporting recovery of undetected cell types is isolation of atypical PB1C4 stromal cells; these progenitors possess adipogenic differentiation potential, but do not show detectable immunostaining of intermediate filaments vimentin or keratin. Given that polymers of intermediate filaments provide strength to the cytoskeleton and protect cells from shear force [45, 46], we speculate that cell shearing may underlie the complexity of PB1C4 cells that was detected by flow cytometry (Figure 2). Clonal populations of long-lived epithelial cells and atypical stromal cells, like PB3B5 and PB1C4, respectively, have not been previously identified in amniotic cell cultures. 

### 4.2. Cell Surface Marker Expression Can Be Uncoupled from Differentiation Potential

Flow cytometry is widely used to characterize stromal cell populations from a variety of sources and coexpression of CD73, CD90, and CD105 is one criterion for MSC identity [18]. Several of our findings suggest that expression of these cell surface markers is not predictive of the differentiation potential of amniotic cells. First, significant proportions of all populations tested expressed CD29, CD44, CD73, and SSEA4, whether or not the populations showed evidence of differentiation potential. Second, both PB1C4 and PB4A2 populations showed osteogenic and/or adipogenic differentiation potential, but the frequency and intensity of CD90 and CD105 expression was very low or, in the case of PB1C4 cells, almost undetectable. Because analysis of BMMSCs was run in parallel in these experiments, low or undetected levels of CD90 and CD105 were not due to technical differences between experiments. Third, PB3B5 epithelial cells and PB4A2 stromal cells (Figure 1) showed almost indistinguishable profiles of surface marker expression by flow cytometry (Figure 2), but only PB4A2 cells showed differentiation potential (Figures 3 and 4). These findings show that adipogenic and osteogenic differentiation potential of amniotic cells can be uncoupled from the cell surface markers that are widely used to gage stromal cell identity and potential differentiation capacity.

Several groups have profiled expression of cell surfaces markers in populations of amniotic cells and tested for differentiation potential to generate connective tissue lineages [23, 29, 43, 47, 48]. It is difficult to compare results from different studies, in part because studies vary in technical methods and in the benchmarks for assigning positive and negative results. Results may also vary because the composition and gestational age of the tested amniotic cell populations vary. Given that the tissue of origin and the conditions used to culture cells are known to impact the differentiation potential of stromal cells [3], the relationship between differentiation potential and expression of cell surface markers may vary for similar reasons. In addition to these influences, future work may show whether expression of cell surface markers and differentiation potential are impacted by paracrine effects on cells in mixed cell populations.

### 4.3. Coexpression of Epithelial and Stromal Cell Characteristics

**Figure 5:**
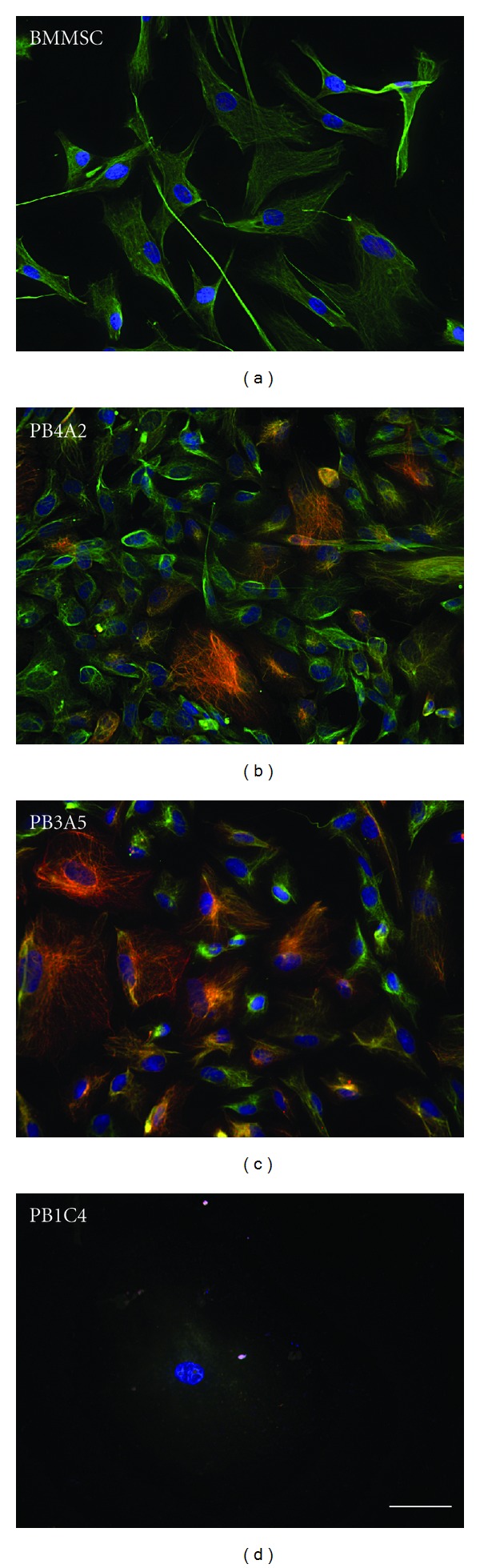
Immunostaining for vimentin and keratin. BMMSCp5, PB4A2p16, PB1C4p12 and PB3B5p11 cell populations were stained for the stromal cell marker vimentin (green), the epithelial cell marker keratin (red) and a fluorescent chromatin dye (blue). Note that virtually all PB4A2 and PB3B5 cells showed keratin staining, although the intensity varied. The clonal population of PB1C4 stromal progenitors did not show immunostaining of prominent networks of either vimentin or keratin. Scale (50 microns) is identical in all panels.

**Figure 6:**
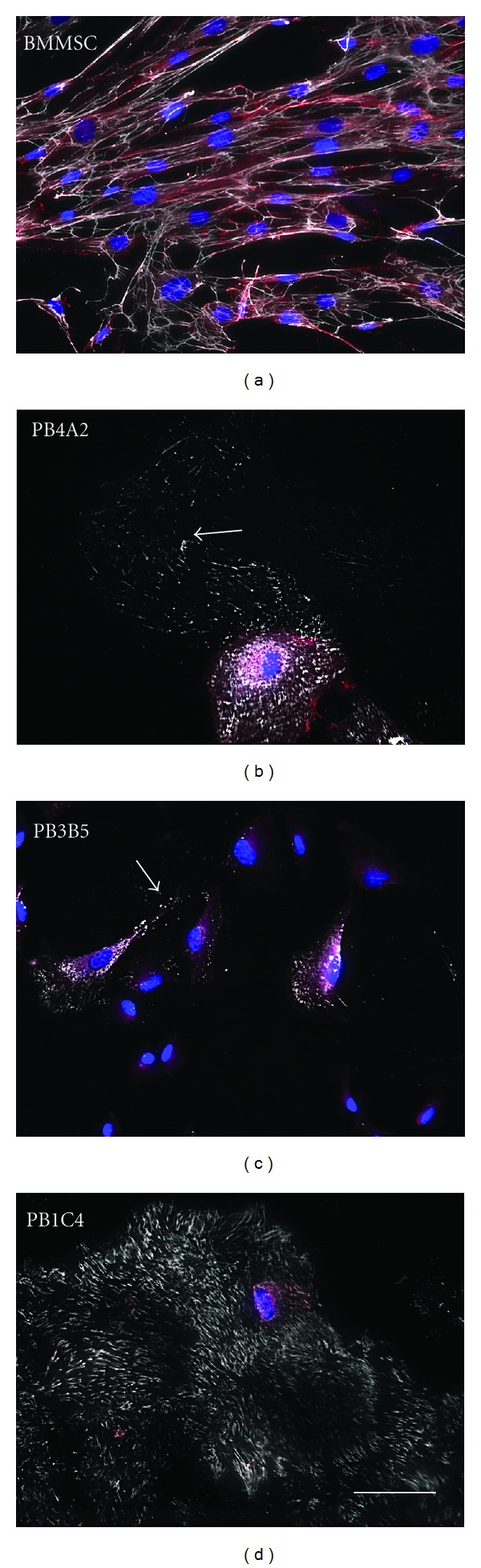
Immunostaining of fibronectin and N-cadherin. BMMSCp5, PB4A2p16, PB3B5p11, and PB1C4p12 cell populations were immunostained for fibronectin (grayscale), N-cadherin (red), and fluorescent chromatin dye (blue). Scale bar, 50 microns, is identical in all panels.

**Figure 7:**
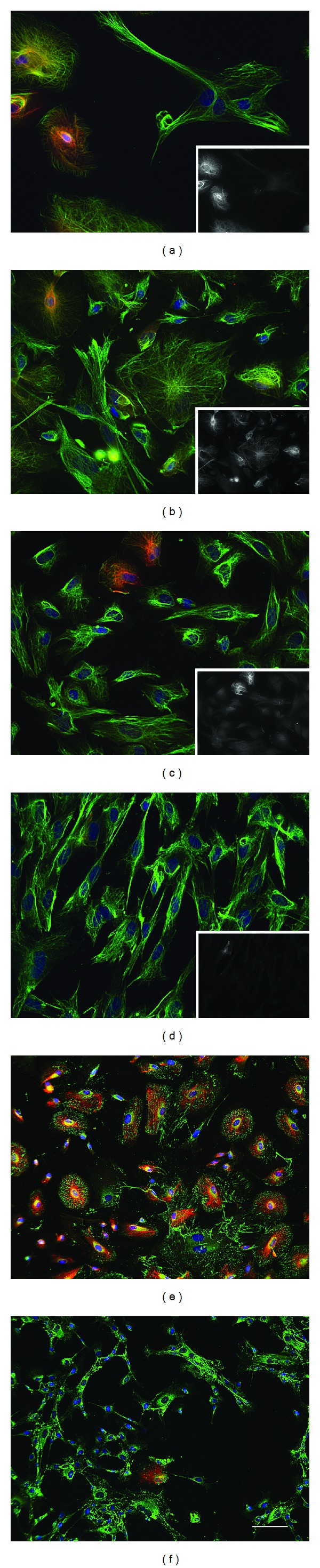
Immunostaining of keratin, vimentin, and fibronectin in mixed cell populations. Mixed cell populations (a, e) ChM1, (b) ChM2, (c, f) ChM3, and (d) ChM4 were immunostained with antibodies against (a–f) keratins (red), (a–d) vimentin or (e, f) fibronectin (green) and (a–f) a fluorescent chromatin dye (blue). Insets in (a–d) show images of keratin signal at low magnification (0.3x). Scale bar, (a–d) 50 microns, (e, f) 100 microns.

Keratin is expressed in epithelial cells and known to be prevalent in amniotic cell populations [34]. High-resolution immunofluorescence imaging showed coexpression of keratin and vimentin in elaborate filament networks in subsets of amniotic cells, including the PB3B5 clonal population of epithelial cells (Figures 5 and 6) and in spherical cells in mixed cell populations (Figure 7) that likely correspond to the epithelial cells detected by phase microscopy. Diversity among epithelial cells is suggested by expression of E-cadherin; this epithelia-specific adhesion molecule was not detected in PB3B5 cells although it was detected in the ChM1 cell population (Table 2). The basis for these apparent differences among epithelial cells is not clear, but differences could reflect variable lifetimes in culture or paracrine signaling in ChM mixed cell cultures that is not present in clonal populations. Differences could also reflect derivation from different fetal sources; fetal skin is a good candidate source of amniotic epithelial cells [34] and it is feasible that amniotic epithelial cells may be derived from placental membranes [49], released naturally or by needle puncture during the amniocentesis procedure. In addition to these sources, epithelia that line the internal surfaces of the fetus are also potential candidates, including epithelial cells from the gastrointestinal tract, lungs, and urinary tracts among others. 

The clonal population of PB4A2 cells showed coexisting epithelial and stromal cell characteristics; PB4A2 cells coexpressed keratin with vimentin and showed multipotential differentiation potential to generate bone and fat. Coexisting epithelial and stromal cell character raises the question of whether stromal cells in amniotic fluid can result from EMT [27, 28]. Precedence for stromal cell derivation through EMT comes from studies in which epithelial cells were induced to release from mammary gland epithelium [50]. These epithelial cells transitioned into MSCs that express stromal cell markers and differentiated into fat, bone, and cartilage [51]. Although derivation by EMT is feasible, it is unlikely to be the exclusive source of stromal cells since PB1C4 stromal progenitors did not show expression of epithelial markers and mixed cell populations included many stromal cell types that lacked keratin expression. Further work is needed to show whether EMT contributes to the pool of stromal cells in amniotic fluid and whether EMT impacts the diversity among amniotic epithelial cells. 

### 4.4. Novel Clonal Populations of Stromal Cells as Resources for ECM Proteins

The extracellular matrix (ECM) is of critical importance to tissue engineering and manufacture of bioengineered organs. Decellularization is perceived to leave behind detergent insoluble ECM that provides form and organization for revascularization and function [52]. While there is considerable advance in tissue and organ engineering, reseeding of decellularized and bioengineered organs with viable, proliferation-competent cells remains a challenge [52–56]. Recent work showed that preseeding biodegradable scaffolds with BMMSCs improved performance of grafted constructs [57]. Within this framework, an outstanding feature of the PB1C4 population was the widespread deposition of fibronectin in low-density cultures (Figure 6). Clonal populations of stromal progenitors like PB1C4 from amniotic fluid offer a cell-based source and/or delivery vehicle for fibronectin and other ECM proteins to improve outcomes with bioengineered scaffolds.

## Figures and Tables

**Table 1:** Independent cell populations from uncultured amniocentesis samples^a^.

Sample	Dilution vol. mL	24 well plate^b^	Clones^c^	Doublets^d^	Senescent clones^e^	Total clones^f^	Total viable clones^g^
RC	25	1	13		1	13	12

LB	25	1	11		2	11	9

GW	100	1	3		1		
		2	2				
		3	3				
		4	6	1		16	14

	125	1	1				
		2	2				
PB		3	4		1		
		4	1				
		5	1			10	9

^a^Amniocentesis samples were donated with informed consent under approved IRB protocol.

^b^Diluted samples were plated in 1 to 5 tissue-culture-treated plates. Clones were assigned unique identifier based on position and plate of derivation.

^c^Populations arising from a single spherical cluster in a single well.

^d^Population derived from 2-well separated spherical clusters in a single well.

^e^Populations that did not continue to proliferate and were discarded after 2 weeks in culture.

^f^Total number of populations obtained from a single amniocentesis sample.

^g^Total number of viable populations expanded and cryopreserved for long-term storage.

**Table 2:** Transcript analysis of E-cadherin and N-cadherin in clonal and mixed cell populations of amniotic cells.

	BMp5	UECp6^b^	PB1C4p6	PB4A2p16	PB3B5p11	ChM1p4
GUSB^a^	1.00	1.00	1.00	1.00	1.00	1.00
CDH1^c^	0.00	30.59	0.00	0.00	0.00	0.82
CDH2^d^	13.22	0.27	16.29	12.98	18.08	23.56

^a^Ct values from 2 replicate TaqMan assays were averaged and normalized to expression of glucuronidase-*β* (GUSB).

^b^Primary cultures of human uroepithelial cells.

^c^Gene expression assay for E-cadherin.

^d^Gene expression assay for N-cadherin.

**Table 3:** Similarities and differences among cell populations.

	Morphology^a^		Differentiation^b^		Contact	Coexpressed markers stromal/	Cadherin^e^	
	Stromal	Epithelial	Fat	Bone	inhibition^c^	epithelial^d^	N	E
PB4A2	+	−	+	+	−	+	+	−
PB1C4	+	−	+	−	−	−	+	−
PB3B5	−	+	−	−	+	+	+	−
BMMSC	+	−	+	+	−	−	+	−
ChM1	+	+	ND^f^	ND	ND	+	+	+

^a^Morphology judged by phase microscopy of cells in newly established cultures. ChM1 mixed cell populations contained both cell types.

^b^On the basis of alizarin red and oil red-O staining of differentiated cell populations.

^c^Nonoverlapping epithelial cells in confluent cultures with cobblestone appearance.

^d^Visible networks of keratin and vimentin in the same cell by high-resolution immunofluorescence microscopy.

^e^Detected with TaqMan gene expression assays and/or by immunofluorescence analysis.

^f^ND: not determined.
